# Surgical Management of a Case of Frontoparietal Fibrous Dysplasia

**DOI:** 10.7759/cureus.88246

**Published:** 2025-07-18

**Authors:** Achraf Moussa, Amine Adraoui, Mohamed Amine Haouane, Adil Ounjar, Ali Akhaddar, Hatim Belfquih

**Affiliations:** 1 Department of Neurosurgery, Avicenne Military Hospital, Cadi Ayyad University, Marrakech, MAR; 2 Department of Pathology, Avicenne Military Hospital, Cadi Ayyad University, Marrakech, MAR; 3 Medical School, Mohammed V University in Rabat, Rabat, MAR

**Keywords:** aesthetic deformity, cranial bone, cranioplasty, fibrous dysplasia, surgical excision

## Abstract

Fibrous dysplasia (FD) is a rare, benign bone developmental disorder that predominantly affects adolescents and young adults. Cranial bones are rarely involved. The definitive treatment for craniofacial FD in adults is complete resection and reconstruction. We present the case of a 20-year-old female with a painless, gradually enlarging scalp swelling in the left frontoparietal region for 10 years. Clinical evaluation showed no neurological deficits or signs of intracranial hypertension. Imaging, including CT and MRI, indicated diploe hypertrophy and a benign large diploic space lesion without intracranial complications. She underwent a craniectomy for mass removal with cranioplasty. Histopathological examination confirmed the diagnosis of FD. The outcome was favorable, with no recurrence at the one-year follow-up. FD is a rare, benign bone disorder characterized by abnormal fibrous tissue replacing normal bone, resulting in weakness and an increased risk of fracture. It can affect various intracranial locations, including the skull bones, the skull base, and the paranasal sinuses. Lesions in the cranial vault may result in visible deformities or functional issues. Diagnosis is based on radiological and histopathological findings, with bone biopsy being the preferred test. Management is primarily conservative, but surgery is indicated for functional preservation and complication prevention.

## Introduction

Fibrous dysplasia (FD) is a benign, non-neoplastic bone disorder marked by the progressive replacement of normal bone and marrow by fibro-osseous connective tissue. It may involve a single bone or multiple bones, and typically follows a slow, progressive clinical course [1,2]. FD cranial bone lesions generally appear as an expanding mass, with symptoms caused by the mass of bone exerted by the lesion. Posterior fossa anomalies have been associated with the pathophysiology of hindbrain herniation, a condition considered to play a key role in the development of syringobulbia. The management of FD may necessitate neurosurgical intervention when definitive treatment is indicated. Nevertheless, surgical intervention in adult cases remains uncommon. While certain non-surgical approaches may slow disease progression, complete surgical excision followed by craniofacial reconstruction is currently regarded as the only curative option for craniofacial FD in adults [3]. We report the case of a 20-year-old female presenting with FD involving the frontal and parietal bones, manifesting as a painless swelling over the left frontoparietal region, who successfully underwent surgical resection of the lesion.

## Case presentation

**Figure 1:**
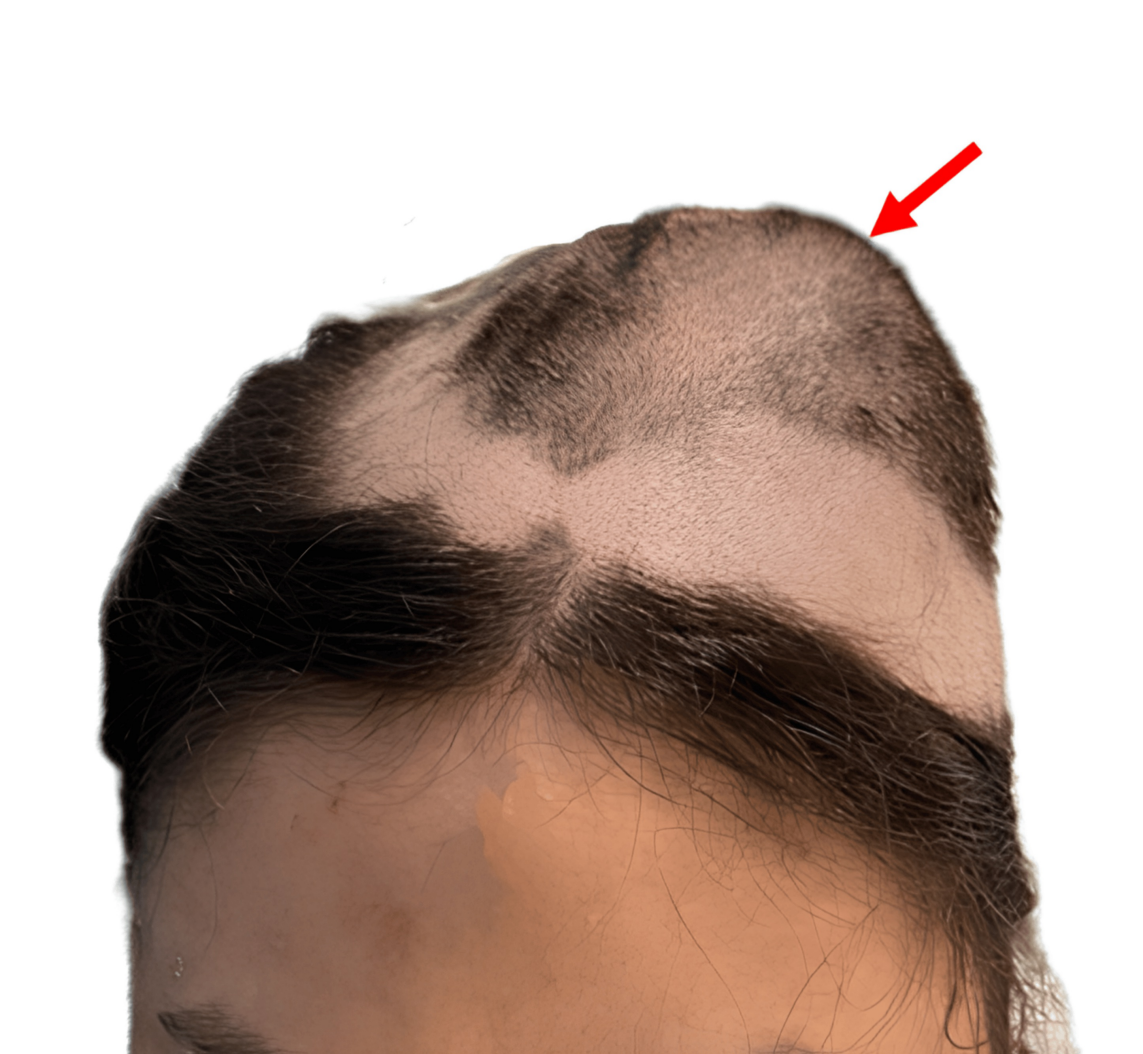
Picture of the patient showing the cranial scalp swelling in the left frontoparietal region (arrow).

**Figure 2:**
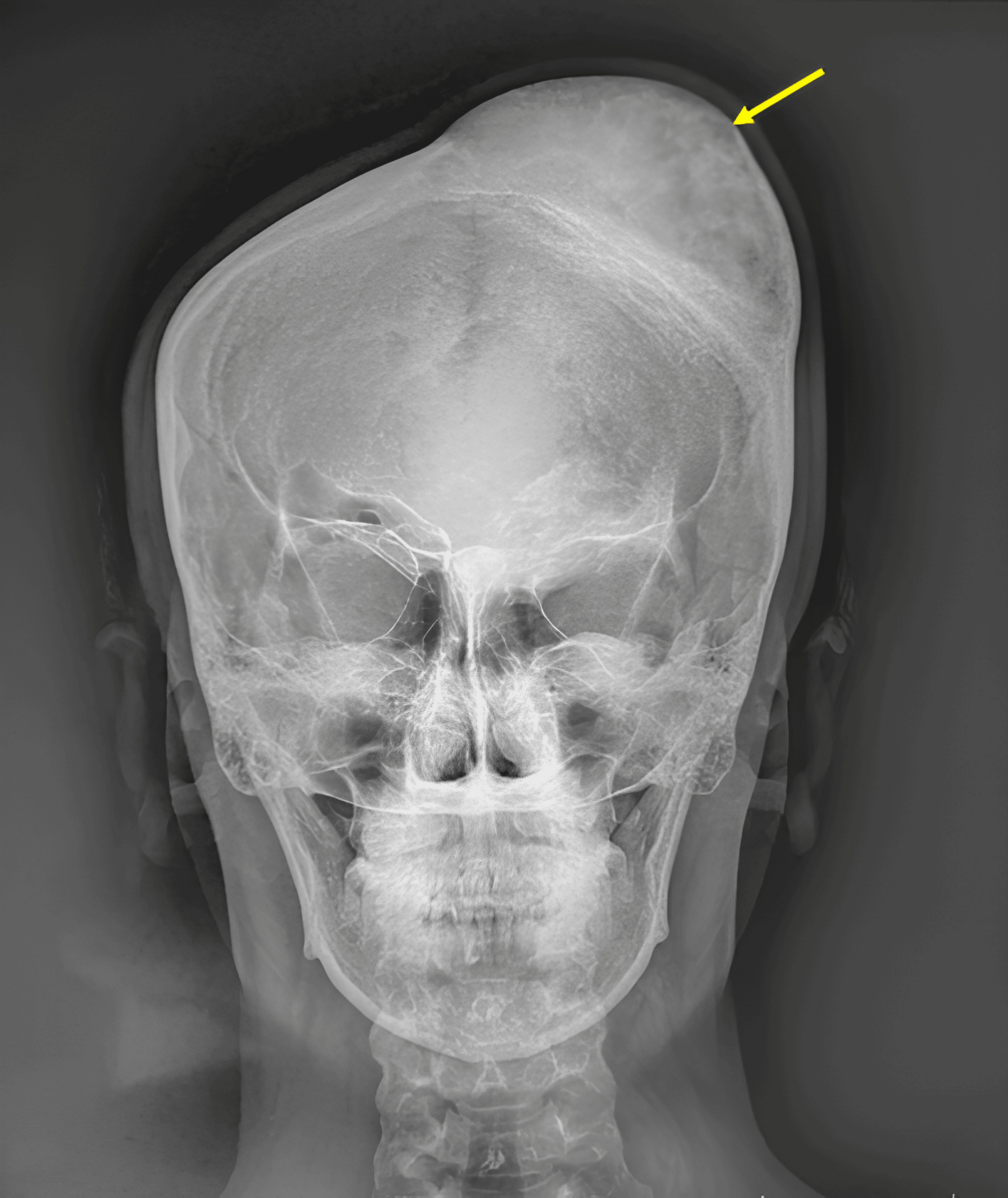
Plain skull X-ray revealing a significant deformation of the vault in the left frontoparietal region (arrow).

**Figure 3:**
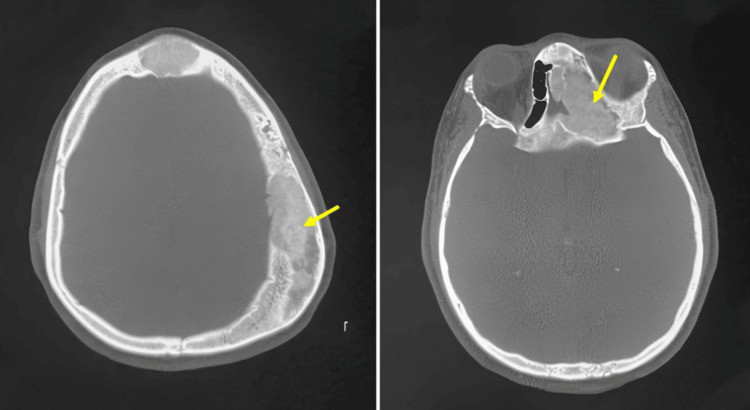
Axial cranial CT scan on bone windows showing a diploe hypertrophy with thinning of the internal and external tables, with a ground-glass appearance involving the frontoparietal bone (arrow) and the frontal and ethmoid sinuses (arrow).

A 20-year-old female presented with a gradually enlarging, painless swelling over the left frontoparietal region, first noticed approximately 10 years before, without nasal and sinus symptoms. The lesion had slowly increased in size over time without nasal, sinus, or neurological symptoms. Clinical examination revealed a conscious patient without signs of intracranial hypertension or general deterioration, no epileptic seizures, and normal neurological examination. Local examination showed a 7 × 7 × 8 cm scalp swelling in the left frontoparietal region (Figure 1), without local inflammation, non-compressible, non-pulsating, and firm consistency without skin spotting. The standard skull X-ray showed a significant deformation of the cranial vault in the left frontoparietal region (Figure 2). Non-CT-scan revealed diploe hypertrophy with thinning of the internal and external tables, with a ground-glass appearance involving the frontoparietal bone and the frontal and ethmoid sinuses (Figure 3).

**Figure 4:**
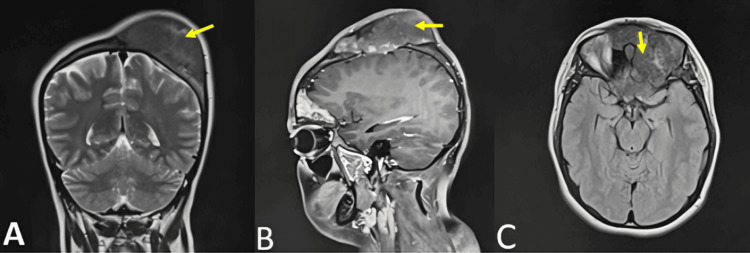
MRI showing a mass with relatively well-defined margins and slight lobulations, centered on the frontal bone and extending to the left parietal, ethmoidal, and sphenoidal bones (arrow). It measures approximately 7 x 7.5 x 6 cm (T x AP x CC). (A) Coronal section on T2-weighted image. (B) Sagittal section on T1-weighted image after gadolinium injection. (C) Axial section on FLAIR sequence. FLAIR, fluid-attenuated inversion recovery

MRI shows a mass with relatively well-defined margins and slight lobulations, centered on the frontal bone and extending to the left parietal, ethmoidal, and sphenoidal bones. It measures approximately 7 x 7.5 x 6 cm (T x AP x CC). The lesion exhibits a heterogeneous T2 hypointensity and shows a heterogeneous enhancement after gadolinium injection on T1-weighted images. This process is responsible for thickening, widening, and cortical blowout of the frontal, parietal, and ethmoidal bones, and the greater wing of the sphenoid bone on the left side. The lesion is also filling the sphenoidal sinus (Figure 4). The phosphorus-calcium tests are normal.

**Figure 5:**
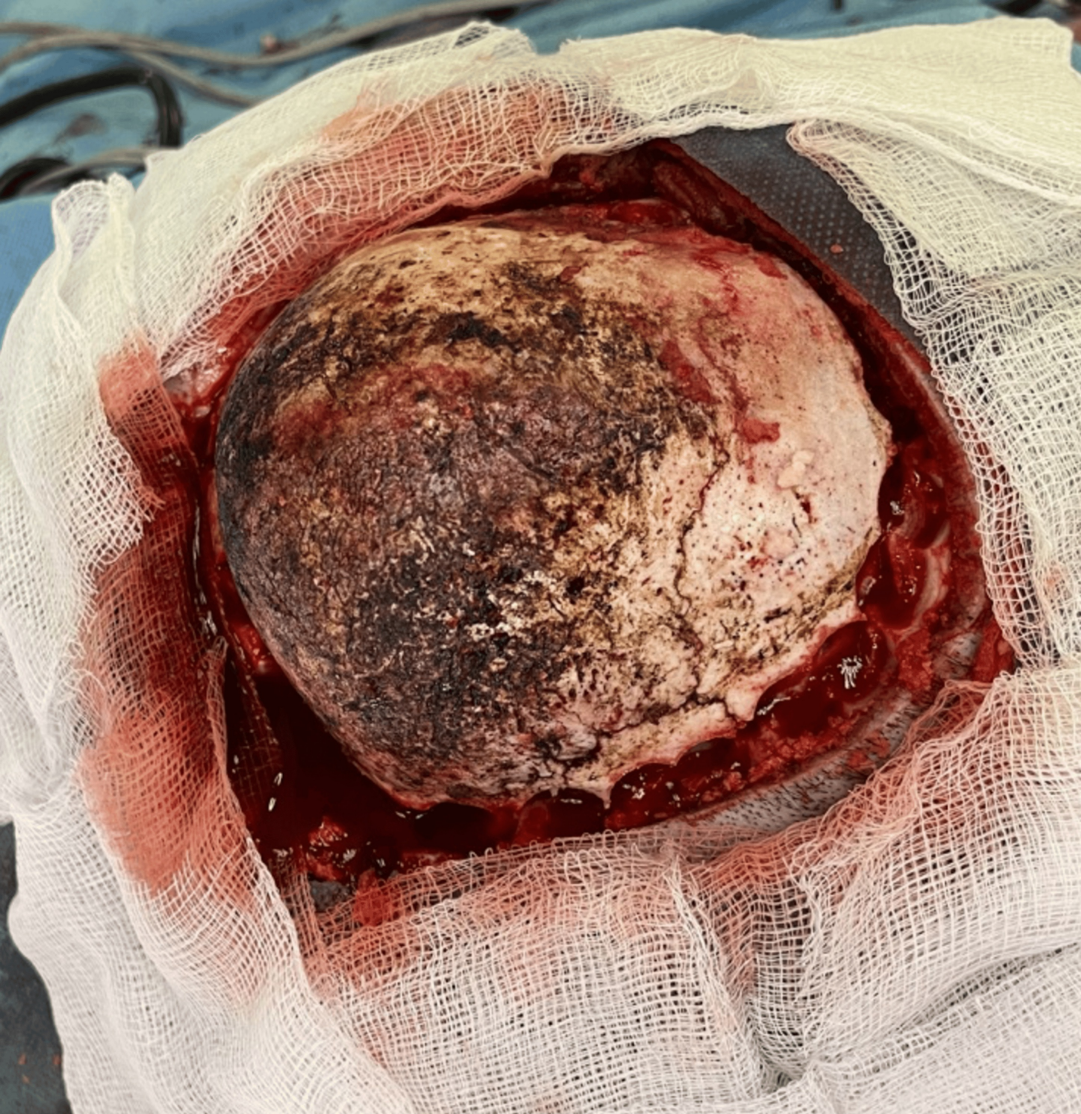
Surgical view showing the craniotomy of the pathological bone using the multiple burr hole technique with more than 25 holes.

The patient was operated on for cranial vault mass removal and histopathological confirmation. Through a left frontoparietal approach beyond the midline, after scalp removal, we performed a craniotomy of the pathological bone using the multiple burr hole technique with more than 25 holes (Figure 5).

**Figure 6:**
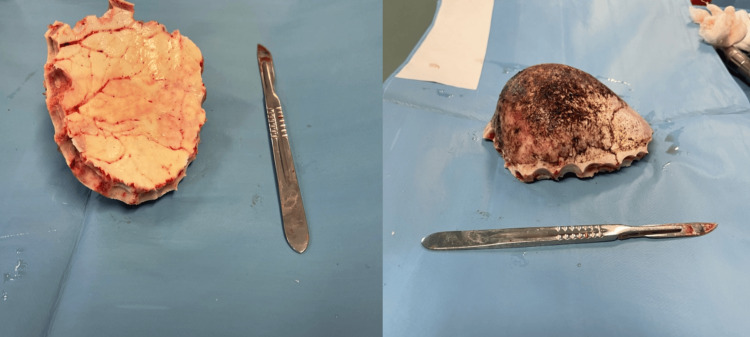
Surgical view showing the specimen bone piece.

**Figure 7:**
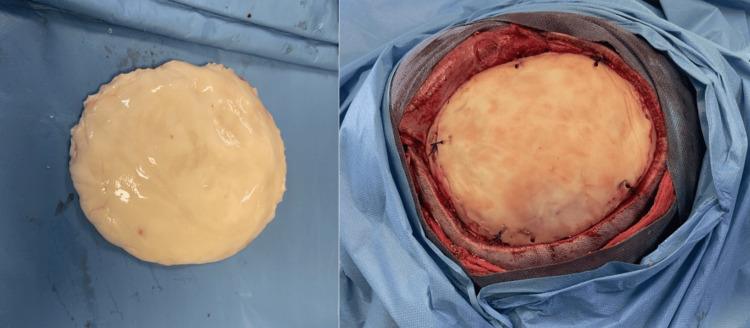
Surgical view showing the cranioplasty.

The specimen was taken as a 10 × 10 × 7 cm bone piece (Figure 6). The dura and cortex were intact. Cranioplasty was performed using methylmethacrylate, which was placed on the skull to replace the pathological bone (Figure 7). An extracranial drain was placed at the end of the surgical procedure.

**Figure 8:**
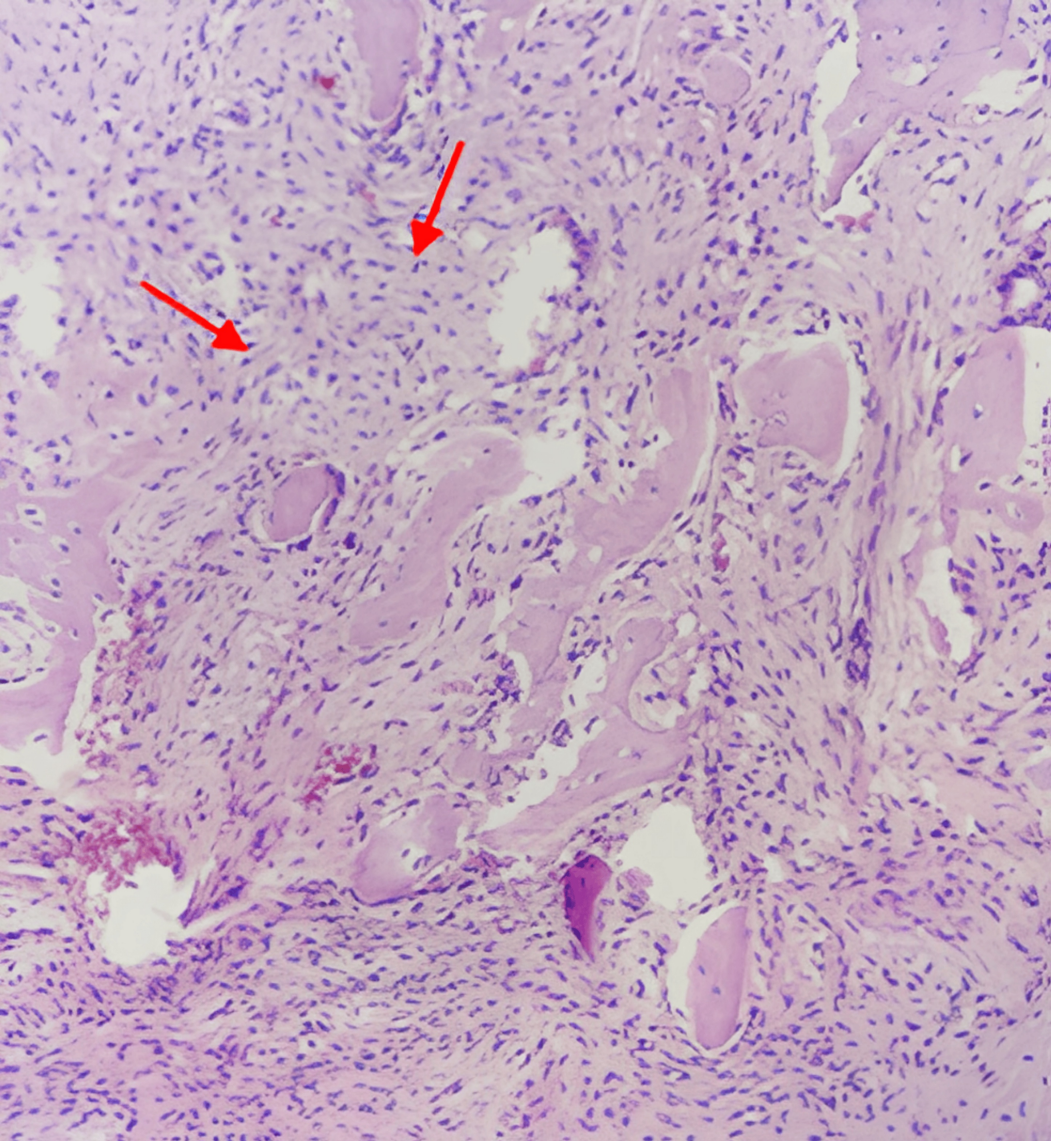
High power view of fibrous dysplasia. Notice the lack of osteoblastic rimming in the bone trabeculae (arrow). The spindle cells present in the stroma are cytologically bland (H&E stain, ×200). H&E, hematoxylin and eosin

**Figure 9:**
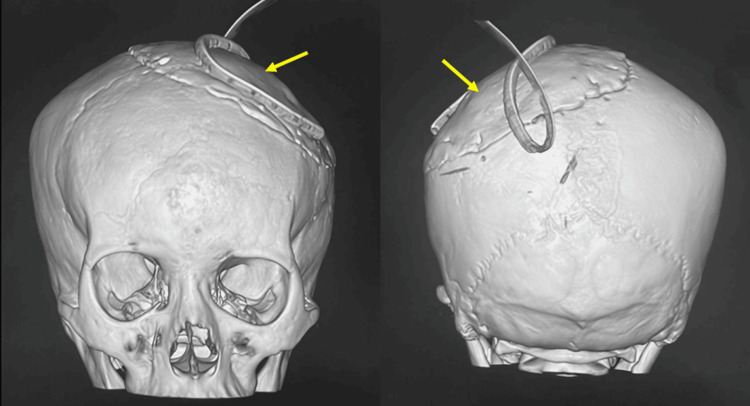
Postoperative 3D CT scan showing the location of the cranioplasty (arrow).

Pathological examination demonstrated irregular trabeculae of woven bone lacking osteoblastic rimming, embedded within a moderately cellular fibrous stroma composed of spindle-shaped cells, without evidence of cytological atypia (Figure 8). These findings were consistent with a definitive diagnosis of FD. The clinical evolution was favorable with the removal of the Redon drain one day after surgery. On control CT scan, there was no extradural collection, the cranioplasty was well placed and fixed without deformity (Figure 9). For the other sites of FD involvement, including the frontal and ethmoid regions, a conservative treatment approach was adopted, as these areas were asymptomatic and posed no functional or aesthetic concerns. The patient was asymptomatic at one-year follow-up after surgical removal without recurrence. 

## Discussion

FD of bone is a rare condition that was initially characterized in 1938 by Lichtenstein as a disorder involving the replacement of normal bone tissue with fibrous tissue. However, its pathological aspects were documented much earlier, in 1891, by von Recklinghausen, who termed the condition "osteitis fibrosa generalisata" [4]. Its prevalence is reported to be less than 1 in 2,000; however, this figure is likely underestimated due to the frequent presence of asymptomatic cases. The condition represents approximately 2.5% of all bone lesions and accounts for about 7.5% of benign bone tumors [5]. Craniofacial involvement often leads to symptoms attributable to the mass effect of the lesion. When expansile lesions involve the bony jugular foramen, they may impinge on adjacent lower cranial nerves, potentially resulting in cranial neuropathies [6].

FD affects both males and females equally; however, some studies have reported a slight predominance in female patients [7]. The present case involves a female patient, consistent with the reported female predilection for FD. The underlying molecular defect in FD is believed to involve a somatic activating mutation in the guanine nucleotide-binding protein alpha-stimulating (GNAS) gene, which encodes the α-subunit of the stimulatory G protein (Gsα). This mutation leads to impaired osteoblast differentiation, fibrous proliferation within the bone marrow, and increased osteoclastic activity, partly mediated by IL-6 overexpression in mutated cells. The mutation may also affect other cell types, resulting in systemic manifestations. When FD is associated with café-au-lait skin macules and endocrine disorders, such as precocious puberty, hyperthyroidism, acromegaly, or Cushing’s syndrome, the condition is classified as McCune-Albright syndrome. Additionally, phosphate-wasting due to fibroblast growth factor 23 (FGF-23) overexpression and the presence of intramuscular myxomas characterize Mazabraud’s syndrome [2].

The diagnosis of bone dysplasia is both clinical and radiological. FD most commonly affects the craniofacial bones, the proximal femur, and the ribs. The monostotic form accounts for approximately 80% of cases, as observed in the present case. Within the craniofacial region, the ethmoid bone is most frequently involved (71%), followed by the sphenoid (43%), frontal bone (33%), and maxilla (29%). Less commonly, the temporal (24%), parietal (14%), and occipital bones (5%) are affected [8]. Two forms have been described, with the monostotic form being 7.6 times more common than the polyostotic form [6]. Because cranial bones are so closely related, especially in the skull base region, extensive lesions across sutures involving several bones are considered monostotic [9], as demonstrated in this case report.

FD presents with characteristic radiographic features that help in its diagnosis. On radiographs, FD lesions are typically classified into three main patterns: cystic, sclerotic, and mixed. The lesions often show a radiolucent ground-glass matrix, particularly in the axial skeleton, and may vary in size and location.

Craniofacial FD typically demonstrates dense and sclerotic lesions as represented in our case. Although endosteal scalloping may be observed, the outer cortical surface typically remains smooth and intact. The lesion may exhibit expansile remodeling as a result of the progressive growth of fibro-osseous tissue. CT-scan is essential in defining individual lesions and evaluating disease extent. It can identify soft tissue masses, bone destruction, and potential malignant transformation.

FD lesions typically show attenuation that usually enhances following intravenous contrast administration. FD lesions on MRI can vary in signal intensity based on the degree of trabeculae, cellularity, and other factors. Typically, FD lesions show intermediate to low intensity on T1-weighted images and intermediate to high intensity on T2-weighted images. After contrast, active lesions exhibit strong enhancement, while inactive ones show milder enhancement. While MRI is not effective in distinguishing FD from other conditions, it can be helpful in evaluating complex cases, such as neurological compression or malignant transformation. Diffusion-weighted imaging is a valuable diagnostic tool that can assist in distinguishing benign from malignant skull lesions [10].

A definitive diagnosis of FD requires histopathological confirmation through a bone biopsy. Radiographically, the most characteristic feature is a "ground-glass" appearance, often associated with a thin cortical margin and poorly defined borders [1].

Pathologies that mimic FD can be classified as other fibro-osseous lesions (such as cementosseous dysplasia and ossifying fibroma), bone cysts, cementoma, Paget's disease, cherubism, hyperparathyroidism, chronic sclerosing osteomyelitis, and osteogenic sarcoma [11].

Management of cranial FD includes a conservative approach, including observation and monitoring, as well as medical and surgical management, which may include surgical remodeling (conservative), radical surgery, and complete removal [12].

Small, asymptomatic lesions that are cosmetically acceptable should be monitored without intervention. Surgical management of lesions in difficult areas of the central skull base, such as the clivus, sphenoid, and petrous temporal bones, is also not recommended due to potential risks. Medical treatments for FD are still being evaluated, focusing on managing abnormal bone formation and associated pain. Current treatments include bisphosphonates such as pamidronate, intravenous calcitonin, and vitamin D and calcium supplements for some patients [12].

Surgery is the preferred treatment for a definitive diagnosis. It allows for the determination of disease progression or malignant degeneration, the removal of compressive lesions for cosmetic purposes, and the correction of failed nonsurgical treatment. The most commonly performed surgical procedure for FD is curettage, followed by bone grafting. This approach involves the thorough removal of the dysplastic bone, followed by reconstruction using autologous bone grafts or synthetic materials such as titanium plates and screws to restore structural stability and contour [13].

In cases where complete excision of the lesion is not feasible due to its size or anatomical significance, contouring of the affected bone to its normal dimensions can be performed using high-speed surgical instruments [14]. More extensive reconstructive procedures involving bone grafting have been associated with lower recurrence rates (approximately 45%) compared to less aggressive remodeling techniques, which show recurrence rates as high as 82% [15]. The primary goals of surgical intervention are to restore structural integrity and function of the involved bone, minimize complications related to lesion location, particularly in the cranial region, and achieve satisfactory aesthetic outcomes.

In our case, we performed an operation for frontoparietal FD. The goal of the surgery was to treat associated symptoms, including bone deformities and aesthetic side effects. The surgical approach consisted of total resection of the left frontoparietal lesion and adaptive reconstruction with cranioplasty to restore appearance. The postoperative outcome was marked by a notable clinical improvement, with no immediate complications, enabling a gradual resumption of daily activities.

## Conclusions

The surgical management of FD in our case resulted in the effective treatment of the aesthetic deformity. The surgical approach, combining resection of the affected bone and adapted reconstruction, offered satisfactory functional and aesthetic results, with a favorable postoperative outcome. This case highlights the importance of surgical management and regular follow-up to optimize results and monitor disease progression.
